# Beware the Muscle: Silent Exercise‐Induced Aminotransferase Elevation as a Diagnostic Pitfall in Hepatology and Pretreatment Screening—A Report of Two Cases

**DOI:** 10.1155/crgm/1771862

**Published:** 2026-10-21

**Authors:** Chebet Chirchir, Antony Kimani, Aaliya Ahmed, Adil Salyani

**Affiliations:** ^1^ Department of Internal Medicine The Aga Khan University Hospital Nairobi Kenya aku.edu

## Abstract

**Background:**

In clinical hepatology practice, pretreatment liver enzyme screening plays a key role in making management decisions, such as initiation of antituberculosis therapy or surveillance of chronic hepatitis B. Unrecognized extrahepatic sources of aminotransferase elevations can lead to misdiagnosis, inappropriate treatment alteration, and unnecessary invasive or costly investigations.

**Case Presentation:**

We present two cases of active young males who were undergoing liver biochemical testing for therapeutic decision‐making for other underlying illnesses. Patient one was undergoing baseline liver enzyme testing prior to first‐line antituberculosis treatment initiation while patient two was undergoing surveillance for e‐antigen‐negative hepatitis B infection. Both patients had asymptomatic aminotransferase elevations mimicking acute hepatocellular injury. There was a marked and disproportionate elevation of aspartate aminotransferase (AST) relative to alanine aminotransferase (ALT), in the setting of otherwise normal cholestatic and synthetic parameters. Targeted history revealed heavy resistance weight training prior to testing. Measurement of serum creatine kinase (CK) confirmed exercise‐induced muscle injury, demonstrating elevated CK concentrations that paralleled the AST trajectory. Cessation of strenuous training led to rapid normalization of aminotransferases and a decline in CK levels, without interruption of tuberculosis management or unwarranted antiviral initiation.

**Conclusion:**

Clinically silent exertional muscle injury is an underappreciated mimicker of acute liver injury. In patients presenting with isolated or disproportionate AST elevation, confirming a myogenic enzyme source via CK quantification can prevent misdiagnosis, eliminate unnecessary liver‐directed workup, and avoid inappropriate modification or initiation of essential pharmacotherapy.

## 1. Introduction

Elevated serum aminotransferases are a signal of hepatocellular injury that can occur secondary to a variety of causes, including infection, drugs, toxins, metabolic, and infiltrative diseases [1]. These enzymes, however, are not confined to hepatocytes, meaning that extra‐hepatic foci of disease or injury could result in an identical elevation. While the magnitude of the rise and the ratio of aspartate aminotransferase (AST) to alanine aminotransferase (ALT) can indicate the primary pathology, other liver parameters like bilirubin, gamma glutamyl transferase (GGT), and muscle‐specific enzymes like creatine kinase (CK) and aldolase are useful in helping clinicians further pinpoint the underlying etiology [2].

In the context of infections like tuberculosis (TB) where pharmacotherapy can lead to hepatotoxicity, guidelines recommend performing a baseline liver biochemical assessment to ensure safe initiation of antituberculous therapy (ATT) and establish a reference point to distinguish any subsequent drug‐induced liver injury (DILI) from preexisting organ dysfunction. Similarly, serum ALT levels form a cornerstone of deciding on the activity of chronic hepatitis B infection, to guide whether antiviral therapy is warranted [3, 4].

Muscle injury is an underappreciated cause of aminotransferase elevation, as muscle is a well‐documented source of both AST and ALT. Asymptomatic CK elevations of > 2000 IU/L can occur following vigorous physical exercise. In one study, CK elevations of > 1000 IU/L led to corresponding rises in AST and ALT in 93% and 75% of patients, respectively [2]. Failure to recognize this entity is associated with numerous investigations for liver disease and poses the risk of deferring highly effective treatment and erroneous therapeutic decisions [5]. We report two cases where alarming elevation of aminotransferases were found during liver enzyme testing for pretreatment screening. Surveillance later revealed that the elevation was due to silent exercise‐induced muscle injury rather than hepatocellular damage, thereby averting gross management errors.

## 2. Case Presentation

### 2.1. Case 1: Extrapulmonary TB

A 28‐year‐old male was referred to the infectious disease (ID) clinic for evaluation of suspected spermatic cord TB following the excision of a painful right spermatic cord mass. Histology of the excised mass demonstrated necrotizing granulomatous inflammation. Ziehl–Neelsen stain was negative for acid fast bacilli. No sample was sent for mycobacterial or fungal cultures.

He was sent for further work up to elucidate the cause of the lesion and get baselines for treatment initiation. Human immunodeficiency virus (HIV) testing, schistosomiasis serology, urine sexually transmitted diseases panel, and chest radiography were all negative or normal. Liver biochemical testing revealed mildly elevated aminotransferases: AST 36.6 U/L (normal 0–34 U/L) and ALT 54.6 U/L (normal 0–49 U/L), with the remainder of the liver biochemical test profile within normal limits. This was assumed to be due to self‐reported moderate alcohol intake which he was advised to stop given planned initiation of anti‐TB therapy. Ten days later, despite stopping alcohol, a repeat liver enzyme panel showed rising aminotransferases (AST 121 U/L and ALT 111 U/L). There was no other drug or medication intake in the intervening period. This prompted an extensive workup for viral hepatitis which was negative.

Upon further history taking, the patient reported engaging in heavy weightlifting at the gym, followed by transient muscle pain that had since resolved but no dark urine. Physical examination showed no muscle tenderness, weakness, or other systemic findings. CK testing was subsequently performed and was markedly elevated at 2255 U/L (normal 46–171 U/L).

The diagnosis was revised to exercise‐induced muscle injury in the background of suspected extrapulmonary TB. He was managed conservatively with oral hydration and cessation of strenuous exercise. Eight days later, repeated testing showed normalization of aminotransferases and a decline in CK to 273 U/L (Table 1), allowing for the safe initiation of empiric anti‐TB therapy (rifampicin/isoniazid/pyrazinamide/ethambutol) per World Health Organization guidelines.

**TABLE 1:** Serial laboratory parameters for Case 1 demonstrating transiently elevated aminotransferases with disproportionate creatine kinase elevation.

Parameter	Reference range	Day 0	Day 11	Day 19
Muscle injury marker				
Creatine kinase (CK)	46–171 U/L	—	**2255** (H)	273 (H)
Hepatocellular enzymes				
Aspartate aminotransferase (AST)	0–34 U/L	**36.6** (H)	**121.0** (H)	23.7
Alanine aminotransferase (ALT)	0–49 U/L	**54.6** (H)	**111.0** (H)	39.3
Cholestatic/biliary markers				
Gamma‐glutamyl transferase (GGT)	0–73 U/L	42.1	43.2	35.5
Alkaline phosphatase (ALP)	46–111 U/L	71.9	68.6	65.9
Synthetic function and bilirubin				
Total bilirubin	5–21 μmol/L	4.5	3.4	5.0
Direct bilirubin	0–5 μmol/L	2.9	2.2	2.9
Albumin	32–48 g/L	43.9	47.2	44.0
Total Protein	57–82 g/L	78.6	78.6	78.5

*Note:* (H) denotes a value above the upper limit of normal; (—) indicates test not performed. Day 0 represents the initial presentation; Day 11 corresponds to the worsening of aminotransferases following exertion; Day 19 represents the recovery period before initiation of anti‐TB therapy. The bold values highlight the values of the elevated aminotransferases and creatine kinase.

### 2.2. Case 2: Chronic Hepatitis B

A 39‐year‐old male was on longitudinal follow‐up for vertically transmitted inactive hepatitis B infection. At initial evaluation one year earlier, investigations confirmed a positive hepatitis B surface antigen with negative hepatitis B e‐antigen, a low viral load (279 IU/mL), and normal aminotransferases (ALT 33 U/L and AST 41 U/L). Liver elastography demonstrated no evidence of fibrosis (5.17 kPa). No antiviral therapy was recommended at the time.

He returned for follow‐up 1 year later after results from an external laboratory done during a routine checkup showed a marked elevation in aminotransferases (AST 589 U/L and ALT 354 U/L), raising concern for a hepatitis flare. However, the synthetic function and cholestatic markers were preserved: Total bilirubin was 12.1 μmol/L (reference range 5–21 μmol/L), GGT was 18 U/L (reference range 0–73 U/L), and alkaline phosphatase was 70 U/L (reference range 46–116 U/L). Hepatitis B viral load testing done concurrently was < 10 IU/mL, effectively ruling out a viral flare. Hepatitis D virus and HIV testing were also negative.

On further interrogation, he reported minimal alcohol consumption and denied the use of any new medications but revealed he engaged in heavy daily weightlifting with fleeting muscle pain. On examination, he was notably muscular with no focal muscle tenderness or weakness. His abdominal examination and other systemic findings were unremarkable.

Repeat aminotransferases and CK levels were assessed and found to be significantly elevated (AST 78 U/L, ALT 64.7 U/L, and CK 1749 U/L). The diagnosis was revised to exercise‐induced muscle injury in the context of inactive hepatitis B carriage. He was managed with oral hydration and cessation of vigorous exercise. Subsequent testing demonstrated rapid improvement in both muscle and liver enzymes (Table 2).

**TABLE 2:** Serial laboratory trends in Case 2 showing elevated aminotransferases with normal cholestatic markers.

Parameter	Reference range	1 year ago (ID clinic)	Day 0 (external lab)	Day 21 (ID clinic)	Day 28 (ID clinic)
Muscle injury marker					
Creatine kinase (CK)	46–171 U/L	—	—	**1749.0** (H)	**468.0** (H)
Hepatocellular enzymes					
Aspartate aminotransferase (AST)	0–34 U/L	41.0 (H)	**589.0** (H)	**78.0** (H)	**37.5** (H)
Alanine aminotransferase (ALT)	0–49 U/L	33.8	**354.0** (H)	**64.7** (H)	43.8
Cholestatic/biliary markers					
Gamma‐glutamyl transferase (GGT)	0–73 U/L	32.5	18.0	19.9	19.7
Alkaline phosphatase (ALP)	46–116 U/L	80.7	70.0	85.4	78.6
Synthetic function and bilirubin					
Total bilirubin	5–21 μmol/L	7.4	12.1	7.2	8.7
Direct bilirubin	0–5 μmol/L	3.6	2.9	4.0	4.6
Albumin	32–48 g/L	46.6	40.0	44.2	45.6
Total protein	57–82 g/L	76.2	63.0	70.8	45.6

*Note:* (H) denotes a value above the upper limit of normal; (—) indicates test not performed. 1 year ago represents the baseline evaluation confirming inactive hepatitis B status; Day 0 marks the acute presentation at an external laboratory showing peak transaminase elevation; Day 21 indicates the follow‐up at the ID clinic capturing the markedly elevated creatine kinase (CK) and resolving aminotransferases; and Day 28 represents biochemical resolution following the cessation of strenuous exercise. The bold values highlight the values of the elevated aminotransferases and creatine kinase.

## 3. Discussion

Evaluating abnormal liver enzymes is a common clinical challenge in outpatient medicine, where elevated aminotransferases are almost universally assumed to indicate acute liver injury and prompt a standard battery of tests for viral hepatitis, autoimmune markers, metabolic disease, or drug hepatotoxicity [1]. However, because aminotransferases are not confined to hepatocytes, extrahepatic injury can produce an identical biochemical picture [2]. In young, active patients, skeletal muscle is an easily overlooked source of enzyme release that can closely mimic primary liver disease [5].

The physiological explanation lies in how these enzymes are distributed across tissues. ALT is mainly cytosolic and is 5–10 times more abundant in the liver than in skeletal muscle [6, 7]. AST, however, is both cytosolic and mitochondrial and is found in high concentrations in liver, muscle, cardiac tissue, and kidneys [5]. Unsurprisingly, following exercise, both enzymes spill into the circulation, but AST rises disproportionately higher than ALT in more than 90% of cases [2]. While both are excellent biomarkers for hepatocellular injury, the burden falls on the clinician’s shoulders to determine whether the focus is a primary liver disease or an isolated muscle injury. Therefore, a high index of suspicion must be maintained, particularly when evaluating muscular young men with an isolated, asymptomatic elevation of aminotransferases in the ambulatory setting. In these scenarios, other liver markers become crucial to strengthen the diagnosis of exercise‐induced muscle injury, as there should be no clinically detectable change in serum bilirubin, ALP, and GGT [8], as precisely mirrored in the biochemical profiles in our two cases.

This exercise‐induced enzyme leak must be distinguished from true rhabdomyolysis, which involves massive muscle necrosis, dark urine, acute kidney injury, and CK levels well over five times the upper limit of normal (> 1000 IU/L) [5, 9]. Neither of our patients met these criteria. They also had no muscle tenderness, weakness, or swelling. Instead, their presentation was clinically silent exercise‐induced muscle injury [10]. Because strenuous weight training can drive aminotransferases up without causing physical discomfort, clinicians will miss the connection unless they explicitly ask about recent gym workouts.

Failing to recognize this mimic leads to expensive workups and bad clinical decisions. In practice, there is often a knee‐jerk reaction to order abdominal imaging, extensive viral serologies, and autoimmune panels [1]. The majorities are negative, and up to half of the hepatobiliary imaging done is normal, or shows incidental steatosis, which only adds to the confusion [8]. The bigger problem is how this affects patient care. In pretreatment evaluations, first‐line anti‐TB therapy may be unnecessarily delayed or switched to less effective drugs due to fear of baseline liver injury [4]. In chronic hepatitis B surveillance, as in our second patient, an exertional rise in aminotransferases can be mistaken for an acute hepatitis flare in an e‐antigen‐negative carrier, leading to unnecessary long‐term antiviral therapy [3]. In clinical trials, spurious enzyme spikes can cause novel treatments to be unnecessarily stopped or participants dropped for presumed DILI [11].

Checking a serum CK is a simple, cost‐effective way to avoid these diagnostic pitfalls. After unaccustomed or heavy weight training, CK typically rises within 48 h, peaks at 72 h, and resolves over 7–12 days [5]. Notably, serum AST demonstrates parallel clearance kinetics, as illustrated by our patients’ serial trajectories in Figure 1 [2, 5, 8]. Therefore, in any physically active individual presenting with an AST‐predominant enzyme pattern and normal canalicular markers, taking a targeted exercise history and checking a baseline CK can rapidly verify an extrahepatic etiology before ordering costly hepatobiliary investigations.

**FIGURE 1:**
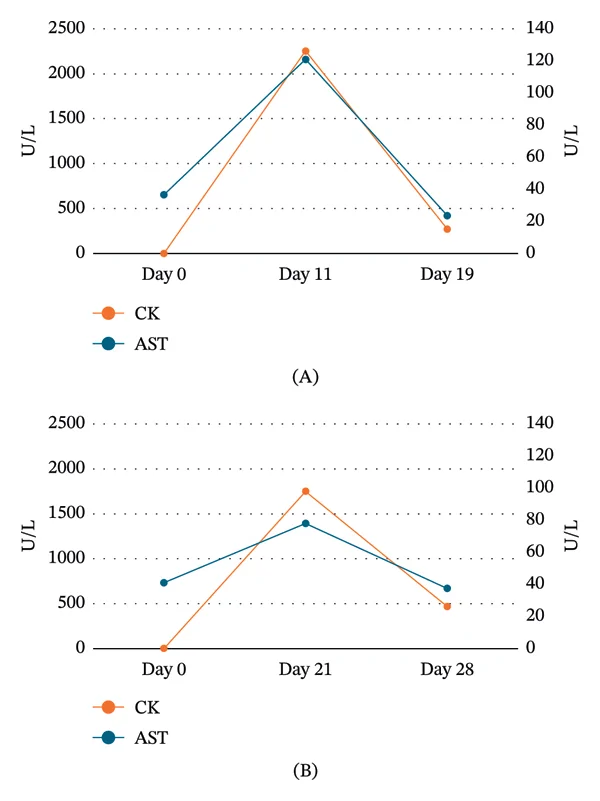
Temporal profiles of serum enzymes demonstrating parallel kinetics in Case 1 (A) and Case 2 (B). In both patients, the trajectory of aspartate aminotransferase (AST, blue line) closely mirrors that of creatine kinase (CK, orange line), confirming the muscular origin of the enzyme elevation. Note that CK is plotted on the primary (left) axis, while AST is plotted on the secondary (right) axis.

## 4. Conclusion

These cases highlight that clinically silent exercise‐induced muscle injury is a mimicker of acute hepatocellular injury and can confound clinical judgment during pretreatment screening and chronic liver disease surveillance. When encountering an isolated or disproportionate AST elevation, especially in active ambulatory patients, clinicians must always remember to consider the muscle. Measuring a serum CK alongside preserved synthetic and cholestatic liver enzymes easily confirms a myogenic source. Ultimately, maintaining this clinical suspicion prevents two major errors: the unwarranted initiation of unnecessary treatments (such as lifelong antivirals) and the dangerous delay or discontinuation of first‐line regimens (like anti‐TB therapy), all while sparing the patient from extensive, invasive, and costly hepatobiliary diagnostics.

## Author Contributions

Chebet Chirchir: conceptualization, clinical data acquisition, literature review, original drafting, critical revision of the manuscript, and final approval.

Antony Kimani: clinical data acquisition, chart review, manuscript editing, and final approval.

Aaliya Ahmed: literature review, data verification, critical manuscript revision, and final approval.

Adil Salyani: conceptualization, clinical oversight, supervisory review, critical intellectual revision of clinical concepts, and final approval.

## Funding

This research received no specific grant from any funding agency in the public, commercial, or not‐for‐profit sectors. The clinical evaluation and publication of this article were performed during the resident authors’ academic training and the supervising author’s employment at The Aga Khan University Hospital, Nairobi.

## Disclosure

All authors read and approved the final manuscript.

## Consent

Written informed consent was obtained from both patients for the publication of this clinical data. All potentially identifying information has been removed.

## Conflicts of Interest

The authors declare no conflicts of interest.

## Data Availability

The clinical data supporting the findings of this report are available within the article and its tables. Further inquiries can be directed to the corresponding author upon reasonable request, subject to strict patient confidentiality and ethical privacy restrictions.
